# Dehydration in Tropical Fevers: Rethinking Fluid and Electrolyte Replacement

**DOI:** 10.7759/cureus.117103

**Published:** 2026-09-28

**Authors:** Nandini Chatterjee, Trupti Gilada, Amol Patil, Harshad Malve, Vijay Chamle, Nikhil Bangale

**Affiliations:** 1 Medicine, Institute of Post Graduate Medical Education and Research, Seth Sukhlal Karnani Memorial (SSKM) Hospital, Kolkata, IND; 2 Infectious Disease and Adult Medicine, Unison Medicare and Research Centre, Mumbai, IND; 3 Medical Affairs, JNTL Consumer Health Pvt Ltd, Mumbai, IND; 4 Medical Safety Sciences, JNTL Consumer Health Pvt Ltd, Mumbai, IND

**Keywords:** chikungunya, dengue, electrolyte imbalance, febrile illness, fluid therapy, malaria, tropical fever, typhoid

## Abstract

Tropical fevers, including dengue, malaria, typhoid, chikungunya, leptospirosis, and scrub typhus, impose a substantial and growing public health burden in the tropical and subtropical regions, particularly the Indian subcontinent. Climate change, monsoon season, and inadequate sanitation further exacerbate transmission dynamics and outbreak patterns. Dehydration represents a critical yet frequently underrecognized complication of these infections. It is attributed to high-grade fever, vomiting, excessive sweating, decreased oral intake, and pathophysiological mechanisms, such as plasma leakage in dengue, hemolysis in malaria, and gastrointestinal losses in typhoid. Electrolyte disturbances, specifically hyponatremia, hypokalemia, and hypochloremia, contribute to the severity of these conditions and likelihood of multi-organ failure. Inadequate hydration amplifies underlying physiological dysfunction, including hemoconcentration, hypovolemic shock, acute kidney injury, and cardiovascular compromise, thereby increasing hospitalization rates, morbidity, and mortality. This review explores the disease-specific hydration challenges across diverse tropical fevers and evaluates the current strategies for effective fluid management. The World Health Organization (WHO), United Nations International Children's Emergency Fund (UNICEF), and national guidelines recommend oral rehydration therapy, formulated with optimized sodium and glucose concentrations to promote rehydration through facilitation of the intestinal sodium-glucose co-transporter (SGLT1) mechanism for management of ambulatory and mild-to-moderate cases. Its efficacy in symptom relief, minimizing intravenous fluid requirements, and accelerating clinical recovery is well established. However, individualized and careful calibration of fluid management remains essential to prevent fluid overload and other related complications. Thus, integrating standardized, evidence-based hydration protocols into routine clinical practice holds the potential to be a practical and cost-effective strategy for managing tropical infection in India and other regions with similar climatic and epidemiological settings.

## Introduction and background

Tropical fevers denote a heterogeneous group of acute febrile illnesses, encompassing dengue, malaria, chikungunya, typhoid, and other infections, that are prevalent in, or unique to, tropical and subtropical regions [1]. The term overlaps with, but is not synonymous with, acute undifferentiated febrile illness, which is characterized by the absence of an identifiable focus of infection at presentation [2]. Tropical fevers pose a substantial public health burden across the globe, reflecting a complex interplay of environmental, climatic, and socioeconomic factors with pronounced monsoon-associated seasonal patterns, affecting millions each year. Rainfall, temperature, and humidity directly influence vector breeding and disease-transmission dynamics [3-6]. The Indian subcontinent, by virtue of its geographical location and climatic conditions, constitutes one of the largest tropical and subtropical regions where these infections remain endemic [1]. Outbreaks are clustered in densely populated urban slums, peri-urban zones, and rural areas with inadequate sanitation and water management [7]. In India, climate change has further intensified the transmission windows by expanding vector habitats and creating prolonged breeding seasons due to altered monsoon intensity and duration, resulting in unprecedented outbreak patterns [6]. The World Health Organization (WHO) reported approximately 96 million symptomatic cases of dengue, 249 million cases of malaria, 9 million cases of typhoid, and 445,271 cases of chikungunya globally in recent times (2023-2025) [8-10]. Dengue and chikungunya remain endemic across India, with transmission risk peaking during the monsoon and post-monsoon seasons. According to the National Center for Vector Borne Diseases (NCVBD), India recorded 233,514 cases of dengue, 255,500 cases of malaria, and 17,930 cases of chikungunya in 2024 [11, 12]. Another recent estimate suggests that approximately 4.9 million typhoid cases occurred in India in 2024 [13].

Dehydration constitutes one of the most critical complications associated with tropical febrile illnesses, arising from multiple pathophysiological mechanisms including high-grade fever, profuse sweating, vomiting, and reduced oral intake [14]. Fluid management thus remains the cornerstone of supportive therapy for tropical fevers, guided by vital signs, hematocrit, and urine output as the mainstay of management [15]. However, the assessment and management of fluid and electrolyte disturbance vary substantially across tropical febrile illnesses. In dengue, plasma leakage during the critical phase produces hemoconcentration and increases the risk of hypovolemic shock and iatrogenic fluid overload, necessitating careful fluid titration based on hematocrit, pulse pressure, capillary refill, and urine output, while avoiding rapid large-volume boluses in the absence of frank shock [16]. Severe malaria was characterized by a sepsis-like syndrome with tissue hypoperfusion, warranting cautious fluid resuscitation that differs from the approach used in dengue [16]. Despite these differences, the heterogeneity in fluid and electrolyte recommendations is often not consistently reflected in supportive-care guidance and is extrapolated from individual diseases, particularly dengue, or from general principles of dehydration, with limited clarity on the applicability in the disease-specific context. Hence, this narrative review aims to examine the hydration challenges associated with tropical fevers and highlight the importance of optimal, disease-specific hydration management, where evidence permits, with appropriate use of oral fluids and electrolytes.

## Review

Literature search

A targeted literature search to identify relevant evidence on dehydration, fluid and electrolyte disturbances, and hydration management in tropical fevers was conducted using electronic search engines such as PubMed/MEDLINE and Google Scholar, supplemented by searches of relevant WHO, Centers for Disease Control and Prevention (CDC), and national/institutional guideline and public health websites, available from 2001 through 2026. The principal search terms and combinations included "tropical fever", "acute febrile illness", "dengue", "chikungunya", "malaria", "typhoid fever", "leptospirosis", "scrub typhus", "dehydration", "fluid management", "fluid therapy", "oral rehydration", "electrolyte imbalance", "electrolyte replacement", "plasma leakage", "acute kidney injury", and related terms. Publications considered relevant encompassed clinical practice guidelines, consensus recommendations, systematic and narrative reviews, observational studies, clinical studies, and other peer-reviewed literature addressing the epidemiology, clinical manifestations, complications, fluid and electrolyte disturbances, and hydration or fluid-management considerations associated with tropical fevers. Evidence was selected and synthesized narratively based on its relevance to the clinical context of the present review.

Tropical fevers and their impact on hydration

As per the Indian Society of Critical Care Medicine (ISCCM), tropical fever not only includes various infections like dengue, scrub typhus, typhoid, malaria, and mixed infections, but also waterborne, food-borne, and vector-borne diseases which are classified within this fever group [1, 17]. Inadequate hydration during tropical fevers results in worsening clinical severity by amplifying key pathophysiological mechanisms such as plasma leakage, hemoconcentration, hypovolemic shock, and cardiovascular collapse, which significantly elevate the risk of severe complications and mortality [18]. The hydration challenges associated with tropical fevers and the consequences of inadequate hydration are discussed in the following sub-sections.

Dengue

During the febrile phase, dehydration (net loss of total body water) is common in dengue due to high fever, persistent vomiting, and poor intake. Consequently, it leads to plasma leakage, which is a distinct pathophysiological process caused by increased vascular permeability during the critical phase, with movement of plasma from the intravascular to the extravascular compartment. When sufficiently extensive, it produces intravascular volume depletion and may progress to hemoconcentration (reduction in effective circulating intravascular volume) and hypovolemic shock, even in the absence of conventional dehydration, necessitating careful fluid titration based on hematocrit, pulse pressure, capillary refill, and urine output, while avoiding rapid large-volume boluses in the absence of frank shock [16, 19].

As the disease progresses, enhanced capillary permeability and plasma leakage cause hemoconcentration, hypovolemia, thrombocytopenia, and risk of shock [20, 21]. Additionally, the vascular hyperpermeability mediated through multiple interacting factors, such as dengue virus non-structural protein 1 (NS1) antigen, endothelial glycocalyx disruption, and activation of immune cells (neutrophils, mast cells, and platelets), results in fluid shifts and organ dysfunction [22]. Symptoms such as headache, myalgia, arthralgia, anorexia, retro-orbital pain, tachycardia, narrow pulse pressure, pleural effusion, or ascites are indicative of fluid imbalance [20]. Dyselectrolytemia is frequently observed in dengue fever. A study found that the most common electrolyte disturbances were hyponatremia and hypokalemia, both of which showed a positive correlation with disease severity. Hyponatremia was 9.7 times more prevalent among dengue patients [23]. A recent observational study has found that patients consuming at least five glasses of fluid demonstrated significantly fewer warning signs, highlighting the protective role of adequate hydration in dengue patients [24].

Malaria

In malaria, fever with sweating, vomiting, and hemolysis are the primary causes of hydration disturbances. Patients with severe malaria typically present with normal cardiac index, vascular resistance, and blood pressure, while renal impairment exacerbates dehydration and electrolyte imbalance, resulting in hemodynamic instability, hypovolemia, and shock, leading to clinical manifestations such as anemia, hepatomegaly, and splenomegaly [25]. Malaria has also been associated with significant electrolyte disturbances, including hyponatremia, hypokalemia, and hypocalcemia, which contribute to disease severity [26]. In *Plasmodium falciparum* infection, complications like acute kidney injury, hydroelectrolytic disturbances, respiratory failure, and shock may develop, reflecting severe intravascular volume depletion and impaired renal clearance. On the other hand, chronic or relapsing malaria, particularly from *Plasmodium vivax* or *Plasmodium*​​​​​​​* malariae*, can aggravate anemia and malnutrition, further worsening hydration status and renal function [25, 27]. Hence, hydration management is critical in malaria, especially in severe cases, as excessive fluid administration during the recovery phase may induce hypervolemia, pulmonary edema, or congestive heart failure, thereby necessitating an individualized, conservative fluid management approach [28, 29].

Chikungunya

Patients with chikungunya typically present with high fever, rash, arthralgia, arthritis, myalgia, backache, and lymphadenopathy [30-32]. A hallmark feature is severe, acute joint pain, accompanied by swelling and stiffness, and a significant proportion of patients develop chronic arthralgia impairing daily function and quality of life. While the disease is often self-limiting, recurrent fever with sweating and vomiting, together with mobility restriction, promotes dehydration, contributing to vertigo, headache, and oliguria [30, 31, 33]. Electrolyte disturbances, including hypocalcemia, hyponatremia, and hypokalemia, exacerbate muscle cramps, weakness, myalgia, joint pains, and neuromuscular dysfunction in patients with chikungunya. Dehydration and electrolyte imbalance may also worsen chikungunya-associated rhabdomyolysis and require aggressive fluid resuscitation [34]. Inadequate hydration further aggravates cartilage and synovial inflammation, intensifying inflammatory responses and potentially delaying recovery, thereby increasing the risk and persistence of chronic chikungunya arthritis and long-term joint dysfunction [33]. Furthermore, dehydration leads to increased rates of hospitalization and prerenal acute kidney injury in patients with chikungunya. A study involving 167 patients with chikungunya indicated dehydration as a potential contributor to chronic chikungunya arthritis (CCA), with early and adequate rehydration associated with reduced risk of CCA (risk difference -16.12, 95% CI, -31.58; -0.66 p = 0.04) [33]. Collectively, these findings suggest that electrolyte deficits, particularly of calcium, potassium, and sodium, substantially amplify musculoskeletal symptoms in chikungunya, highlighting the importance of adequate electrolyte-replete hydration for neuromuscular function and recovery.

Typhoid and Other Febrile Illnesses

The other febrile illnesses, including typhoid fever, scrub typhus, and leptospirosis, are also frequently associated with substantial hydration challenges due to persistent high-grade fever, gastrointestinal fluid losses (vomiting and diarrhea), and reduced oral intake, predisposing patients to fluid and electrolyte imbalance. Dehydration in these conditions results in fatigue, headache, myalgia, and abdominal pain and aggravates renal and hepatic injury, increasing the risk of multi-organ failure [24, 35-39]. A prospective observational study conducted among 136 patients with typhoid documented dehydration among 15.4% of patients [40]. In contrast, another investigation found electrolyte abnormalities in 60 patients suffering from typhoid fever wherein 51% reported hypochloraemia, 50% hyponatremia, and 36% hypokalaemia [41].

Collectively, these findings highlight the pivotal association of dehydration across tropical infections, reinforcing the need for timely recognition and proactive, tailored fluid management to prevent complications, reduce organ dysfunction, and improve overall clinical outcomes. The clinical consequences associated with inadequate hydration across tropical illnesses are presented in Table 1. The growing impact of climate change, especially recurrent heat waves, may further amplify the incidence and severity of these infections by altering vector ecology and increasing dehydration risk [42]. 

**Table 1:** Clinical consequences of poor hydration in common tropical illnesses CCA - chronic chikungunya arthritis

Tropical fever	Consequence	Clinical implication
Typhoid	Leads to fluid and electrolyte losses and abnormalities, including hyponatremia, hypokalemia, and hypochloremia [35]	Increased risk of dehydration-related complications, electrolyte imbalance, weakness, and prolonged illness [41]
Malaria	Causes or aggravates hypovolemia and contributes to impaired renal perfusion [25]	Increased risk of acute kidney injury, renal impairment, and adverse clinical outcomes; careful fluid management required to avoid both dehydration and fluid overload [28, 29]
Dengue	Exacerbates plasma leakage, hemoconcentration, hypovolemic shock, and cardiovascular collapse; Dyselecrolytemia [18, 23]	Increased disease severity, higher risk of dengue shock syndrome, mortality [20, 21]
Chikungunya	Associated with increased hospitalization rates and prerenal acute kidney injury; aggravation of chronic chikungunya arthritis [43, 44]	Greater risk of acute complications and chronic chikungunya arthritis; delayed recovery and prolonged morbidity [33]

Guidelines and recommendations for hydration management in tropical fevers

Hydration management in tropical fevers should be guided by disease-specific protocols emphasizing early assessment, tailored fluid therapy, and vigilant monitoring. Dengue guidelines focus on early recognition of plasma leakage and recommend encouraging oral fluids or oral rehydration therapy in ambulatory cases while reserving IV isotonic crystalloids for critical phases with close monitoring to avoid fluid overload [1, 20, 45-47]. On the other hand, malaria guidelines emphasize conservative fluid administration (oral rehydration when feasible and conservative IV therapy in severe cases) to prevent pulmonary edema while maintaining organ perfusion, with special pediatric considerations for resource-limited settings [48]. Supportive care for chikungunya and typhoid centers on oral hydration with electrolyte replacement, reserving IV fluids for severe dehydration or oral intolerance; leptospirosis similarly demands careful fluid-electrolyte management, with IV therapy tailored to renal function [35, 49-52]. Table 2 summarizes the international and national guidelines for effective management of these acute febrile illnesses.

**Table 2:** Guidelines and recommendations for hydration management in tropical fevers API - Association of Physicians of India; CDC - Centers for Disease Control and Prevention (United States); F-IMNCI - facility-based integrated management of neonatal and childhood illness; ISCCM - The Indian Society of Critical Care Medicine; IV - intravenous; NCVBDC - National Center for Vector Borne Diseases Control (India); ORS - oral rehydration solution; WHO - World Health Organization

Tropical fever	Agency	Recommendations/ Guidelines
Typhoid	API	Supportive measures with oral or intravenous hydration and correction of fluid-electrolyte balance [35, 50].
	F-IMNCI	Supportive measures, including oral or intravenous hydration and appropriate nutrition.
Malaria	WHO	Cautious and individualized fluid management, maintaining adequate hydration while avoiding fluid overload, guided by careful monitoring of perfusion and urine output [48]. Careful, monitored fluid resuscitation, balance needs to correct hypovolaemia while avoiding pulmonary/cerebral oedema; haemodynamic monitoring and urine output guided therapy.
Dengue	WHO	Encourage oral intake of ORS, isotonic electrolyte solution, fruit juices, barley or rice water and continue oral fluid and electrolytes from day 1 for patients who can drink, while avoiding plain water and with caution to prevent overhydration, particularly in infants and young children [20, 45] Administration of IV isotonic crystalloids or colloid solution for the severe or critical (plasma leakage) or shock phase, with close monitoring [20].
	CDC	Stay well-hydrated [47]
	NCVBD	Encouraged to maintain adequate hydration by drinking plenty of fluid (mild cases) and IV isotonic crystalloids (moderate cases) or colloid infusion (refractory shock with plasma leakage) [46].
	ISCCM	Isotonic fluid infusion during plasma leakage [1].
Chikungunya	WHO	General rest and drinking plenty of fluids [52]. Supportive management, including adequate oral fluid intake to prevent dehydration, especially in patients with high fever or poor appetite [49].
	CDC	Rest and adequate intake of fluids
	NCVBDC	Supportive care with fluid supplementation or intravenous fluids, as required, to maintain hydration.
Leptospirosis	WHO	Maintain fluid and electrolyte balance with guided oral or IV hydration based on clinical assessment. Ensure adequate urine output. Use cautious IV fluids in renal involvement or hypotension to avoid fluid overload [51].

Fluid and electrolyte replacement in tropical fevers

Hydration Assessment and Treatment Monitoring

Assessment of hydration status should be an integral component of the initial evaluation and monitoring of patients with tropical fevers. It should combine the clinical history of fluid intake and ongoing losses with thirst, mucosal dryness, skin and peripheral perfusion, capillary refill time, pulse rate and blood pressure, body weight where feasible, and urine output, supplemented by serum electrolytes, renal function, hematocrit, and other investigations according to disease severity and clinical context. In dengue, particular attention is required to distinguish simple dehydration from intravascular volume depletion caused by plasma leakage; serial hematocrit, pulse pressure, capillary refill, vital signs, urine output, and clinical signs of fluid overload can help guide the fluid therapy [20, 53]. In severe malaria, fluid replacement should be individualized because hypovolemia may coexist with microcirculatory impairment, acute kidney injury, or pulmonary edema; therefore, urine output, blood pressure, respiratory status, and overall clinical response should be monitored closely, with avoidance of unnecessary rapid or excessive intravenous fluid administration [29, 54]. For typhoid and other febrile illnesses, hydration should similarly be guided by the severity of dehydration, oral intake, and ongoing losses, while treatment of the underlying infection remains central to recovery [49, 53, 55].

Rehydration therapy should therefore be viewed as a dynamic process rather than a fixed fluid prescription: oral fluids containing appropriate electrolytes should be used whenever clinically appropriate, whereas isotonic intravenous crystalloids may be required for patients unable to maintain oral intake, with significant dehydration, circulatory compromise, persistent vomiting, or severe disease. Response to therapy should be reassessed through improvement in thirst and symptoms, peripheral perfusion and hemodynamic parameters, adequate urine output, stabilization or normalization of relevant laboratory indices, and absence of fluid overload; intravenous fluids should be reduced or discontinued once adequate oral intake and effective circulation are restored, particularly in conditions such as dengue and severe malaria [1, 49].

Intravenous Fluid Therapy

Electrolyte imbalances represent a significant concern in tropical fever cases, with hyponatremia being the common electrolyte disturbance with potential for adverse clinical outcomes if left unaddressed [23, 25, 41, 56]. Intravenous (IV) fluid therapy remains the standard approach for patients with tropical fevers who have severe dehydration, hemodynamic instability, or persistent vomiting that limits oral intake. It enables rapid and controlled delivery of fluids and electrolytes, making it particularly valuable in critical situations such as dengue shock or severe electrolyte disturbances [11, 19, 57]. However, IV therapy is associated with procedural complications including phlebitis, healthcare-associated infections, increased costs, and nursing burden [58]. Excessive or poorly monitored administration may also result in fluid overload, a major concern in dengue due to capillary leak syndrome [19].

Oral Rehydration Therapy

Global and national guidelines, including the WHO, United Nations Children's Fund (UNICEF), and the Indian Academy of Pediatrics, recommend oral rehydration therapy for management of febrile illnesses [20, 30, 31, 45-49, 52, 53, 59]. Real-world experience from resource-constrained settings and emergency scenarios has further reinforced the practical utility of oral rehydration therapy ORT as a viable substitute for IV fluids in managing volume and electrolyte deficits [60]. For patients with mild-to-moderate dehydration who can tolerate oral intake, oral electrolyte solutions provide an effective alternative. Compared with IV therapy, oral rehydration is less expensive, associated with fewer hospital admissions, shorter hospital stays, and comparable correction of fluid and electrolyte deficits. It has been shown that patients with fever demonstrate notable clinical recovery when managed with oral rehydration solutions, highlighting the therapeutic importance of these oral rehydration solutions in tropical fever management [61].

A range of oral rehydration drinks, some even ready-to-drink solutions, are available depending on patient needs, preferences, and clinical scenarios. Among these, oral rehydration solution (ORS) containing electrolytes and glucose is a validated option for replacing fluid and electrolyte losses associated with fever and vomiting. Adequate oral intake of ORS may help restore hydration and potentially reduce the need for hospitalization, particularly in patients with dengue [20, 45]. Additionally, isotonic electrolyte solutions, characterized by osmolality comparable to plasma and containing balanced levels of key electrolytes such as sodium, potassium, and chloride, offer a complementary hydration strategy by facilitating efficient rehydration through rapid fluid absorption [62, 63]. They also effectively replenish extracellular fluid and support restoration of circulating blood volume [62]. The inclusion of glucose in these oral rehydration formulations is physiologically essential, as it activates the sodium-glucose co-transporter (SGLT1), promoting coupled sodium and water absorption across the intestinal epithelium, ensuring rapid plasma volume restoration without causing electrolyte imbalance [64, 65]. Thus, oral rehydration therapy offers a simple, robust approach to correcting dehydration and electrolyte loss in tropical infections.

However, the palatability of ORS remains a major concern, particularly in tropical fevers, where reduced appetite, fatigue, and gastrointestinal symptoms limit voluntary fluid intake, highlighting the importance of taste in enhancing patient acceptance and adherence [24, 35-38, 49, 66, 67]. The incorporation of non-nutritive sweeteners such as sucralose, within permissible limits (acceptable daily intake of 15 mg/kg body weight), has been approved even by the WHO to improve the taste of oral electrolyte drinks without increasing caloric or glycaemic burden [68-71]. The improvement in palatability of oral electrolyte formulations has the potential to enhance fluid intake, facilitate early correction of dehydration, and contribute to improved clinical outcomes in patients with tropical febrile illnesses.

Ready-to-Drink Electrolyte Solutions

The Association of Physicians of India's recent guidelines recommend that oral fluid, electrolyte, and energy management should be prioritized in hospitalized patients with non-diarrheal diseases to prevent dehydration-related complications, which have been associated with prolonged hospital stays and increased mortality rates. The Association of Physicians of India recommends that ready-to-drink (RTD) electrolyte solutions are an available and effective rehydration strategy during transition care and at discharge [72]. The concept of IV-to-oral de-escalation, transitioning patients from parenteral to enteral fluid therapy as clinical status permits, is gaining recognition as an important strategy in tropical fever management. RTD formulations are also available, which are premixed, standardized, palatable, and especially advantageous in this transitional phase, eliminating the need for reconstitution and improving adherence. Taken together, the available evidence supports a stepwise, clinically guided approach: IV fluids should be initiated when oral intake is not feasible, or dehydration is severe, but should be systematically de-escalated to oral electrolyte solutions as soon as the patient's clinical condition permits, thereby reducing IV-associated complications, lowering costs, promoting earlier mobilization, and supporting the continuum of rehydration care through to discharge [72]. However, while the benefits of rehydration therapy are well-established, caution is warranted regarding excessive fluid administration. Some concerns were raised, particularly in dengue patients, where high-volume fluid administration may paradoxically increase the risk of severe complications [73]. This evidence highlights the importance of individualized, carefully calibrated fluid management strategies tailored to specific patient needs and clinical contexts.

## Conclusions

Optimal hydration and electrolyte management are central to improving outcomes in tropical fevers. Evidence supports low-osmolarity fluid electrolyte drinks as a safe, cost-effective intervention that enhances fluid absorption, prevents electrolyte imbalance, and reduces complications across diverse febrile illnesses. Integrating standardized, disease-specific rehydration protocols into routine clinical practice strengthens early management and minimizes progression to severe disease. Ongoing training of healthcare professionals, patient education, and adherence to WHO and national hydration guidelines can significantly reduce febrile illness-related morbidity and mortality. Therefore, strengthening hydration-focused strategies represents a practical, evidence-based approach to improving recovery and resilience in populations affected by tropical infections.
